# Outcomes of Kidney Transplants From Toxoplasma-Positive Donors: An Organ Procurement and Transplant Network Database Analysis

**DOI:** 10.3389/ti.2024.13203

**Published:** 2024-07-11

**Authors:** Lavjay Butani, Daniel Tancredi

**Affiliations:** ^1^ Department of Pediatrics, University of California Davis Medical Center, Sacramento, CA, United States; ^2^ Department of Pediatrics and the Center for Healthcare Policy and Research, University of California Davis Medical Center, Sacramento, CA, United States

**Keywords:** toxoplasma, kidney, outcomes, survival analysis, infection

## Abstract

There is a need to reconsider the acceptance of organs from donors considered suboptimal, in the absence of data. Toxoplasma antibody-positive donors (TPD) constitute one such group. The objective of our study was to compare graft survival in deceased donor renal transplant (Tx) recipients, stratified by Toxoplasma IgG status, using the Organ Procurement and Transplantation Network (OPTN) database. A log-linear event history regression model for graft failure categorized by Toxoplasma IgG status, adjusting for confounders was applied to first kidney-only Tx recipients from 2018 to 2022. Of the 51,422 Tx, 4,317 (8.4%) were from TPD. Acute rejection and graft failure (5% each) were similar between groups. Crude graft failure was 7.3 failures per 100 person-years for TPD recipients compared to 6.5 failures per 100 person-years for the Toxoplasma-negative group (*p* 0.008). The crude failure rate ratio was 1.14 with an adjusted hazard rate ratio of 1.04 (95% CI: 0.94, 1.15, *p* 0.39). In renal Tx recipients, TPD graft recipients have comparable survival to Tx from Toxoplasma-negative recipients. While caution and close monitoring of recipients post-Tx for surveillance of disseminated toxoplasmosis are still warranted, our study suggests that patients can be successfully managed using TPD organs.

## Graphical Abstract

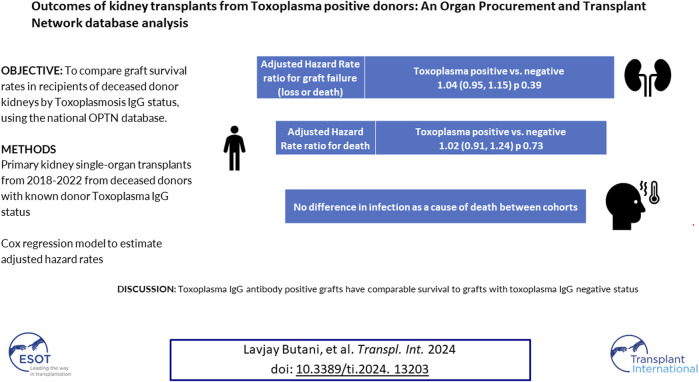

## Introduction

Given the marked shortage of organ donors relative to the number of patients on the waiting list, it behoves the Tx community to systematically review organ acceptance practices which may be based on historical data and anecdotal experience. Moreover, with increasing experience with Tx techniques and management, organ donors previously considered unsuitable for Tx, may no longer be so; examples of such instances include the current practice of utilizing kidneys from donors after circulatory arrest [1], and from donors who have experienced acute kidney injury [2].

Toxoplasma positive donors (TPD) are another such organ donor group, from which Tx has been considered risky and discouraged, based on historical data demonstrating high mortality, especially in heart transplant recipients [3]. Toxoplasma is an intracellular protozoan parasite that is common in humans and animals and that causes mild and self-limited illness in immunocompetent individuals [4]. In its cystic form, it remains latent in various tissues after infection, such as the heart, and can be transmitted through the Tx of such organs. Moreover, Toxoplasma can get reactivated in immunosuppressed states and lead to life-threatening and potentially fatal illnesses [4]. In 2017, based on data from heart Tx recipients, the OPTN issued an advisory and mandated the screening of all deceased organ donors for Toxoplasma, with suggested guidelines for the acceptance of such organs leaving the final decision to individual Tx centers^1^.

The goal of our study was to compare outcomes in recipients of TPD renal Tx, to those who received organs from Toxoplasma-negative donors, in a contemporary cohort of patients following the OPTN policy change, in light of advancements in the diagnosis, prevention and treatment of infections in patients receiving Tx. To our knowledge, there are no published data addressing the frequency of TPD organ acceptance in Tx recipients or their outcomes in the current era.

## Patients and Methods

We conducted a retrospective cohort analysis of the OPTN database, to identify Tx recipients who had received their first deceased donor (DD) kidney-only Tx between 28 February 2018, and 30 June 2022. Donors and recipients who tested positive for HIV were excluded from the analyses because of their higher risk of toxoplasmosis. To be included in the study we also required that the recipient have a graft that had not failed on the day of the surgical procedure in order to be able to analyze our primary outcome measure (time to graft failure or death) using survival analyses. Data on donor and recipient demographics and peri-Tx characteristics were compared among Tx recipients stratified by Toxoplasma IgG antibody status (positive or negative), using Pearson Chi-squared or Fisher exact tests for categorical variables and the Kruskal Wallis test for continuous variables. Kaplan-Meier curves were estimated and categorized for the primary outcome, time to graft failure (graft loss or patient death) by Toxoplasma antibody status. For the primary outcome measure, the following recipient, donor, and Tx-related characteristics were included in the multivariate models-recipient age (pediatric <18 years at the time of Tx) and sex, self-reported race-ethnicity, cause of chronic kidney disease (CKD), donor age and cause of death, OPTN region where the Tx occurred, donor source, need for pre-Tx dialysis, number of HLA mismatches, year of Tx, pre-Tx hypoalbuminemia, and cold ischemia time (CIT). Secondary outcome measures were rates of DGF (defined as the need for dialysis in the first week after Tx), treatment for AR at 1 year, and causes of graft loss/death. We also performed separate univariate and multivariate analyses on outcome measures using a pediatric-only (<18 years) recipient dataset since the pediatric population is at the greatest risk of complications from TPD Tx, due to the lower seroprevalence of toxoplasma in children [5], increasing their risk of developing *de novo* disease.

Datasets were assembled and analyzed using Version 9.4 of SAS (Cary, NC), with multivariate analysis for time to graft failure modeled using the Cox proportional hazards regression procedure. All available data were used, and the resulting precision of the estimates is reflected by the width of the confidence intervals.

The study was granted exempt status by the University of California Davis Institutional Review Board.

## Results

### Descriptive Data

During the study period, 51,422 patients received a DD renal Tx; baseline characteristics of the study subjects are displayed in detail in Table 1. The majority of the recipients were adults (95.7%), men (59.9%), self-identified as non-Hispanic white (34.6%) and black (34.4%), and had glomerular disease as the cause of their CKD (77.3%), representative of the larger Tx population. The leading cause of death was anoxia (51.7%) followed by head trauma (29.4%). The majority of Tx were poorly matched for HLA antigens (as expected for DD Tx), with 76.8% of Tx having >3 HLA mismatches. The majority of patients had received pre-Tx dialysis (90%). Only a small fraction of the study population received TPD organs (4,317; 8.24%). The rates of DGF, and AR at 1 year, were 28.5% and 4.9% respectively.

**TABLE 1:** Demographic and Tx characteristics of study subjects (n 51,422).

Demographic and Tx-related characteristics	Frequencies
Recipient sex (female)	20,599 (41%)
Recipient age (pediatric)	2,166 (4.2%)
Recipient race-ethnicity	Black 17,679 (34.4%)
	Non-Hispanic White 17,797 (34.6%)
	Hispanic 10,732 (20.9%)
Recipient cause of CKD	Glomerular 36,204 (70.4%) Structural 5808 (11.3%)
Donor Toxoplasma IgG status (positive)	4,317 (8.4%)
Donor cause of death	Anoxia 26,571 (51.7%) Head trauma 15,124 (29.4%) Stroke/Cerebrovascular 7,764 (15.1%)
HLA mismatch	02,151 (4.2%) 1,426 (0.8%) 22,198 (4.3%) 37,170 (13.9%) 414,536 (28.3%) 517,291 (33.6%) 67,650 (14.9%)
Receipt of pre-Tx dialysis	46,295 (90%)

CKD, chronic kidney disease; HLA, human leukocyte antigen; Tx, Transplant.

### Comparing TPD and Toxoplasma-Negative Donor Cohorts

The two cohorts were comparable in most demographic and Tx characteristics with a few exceptions, as outlined in Table 2. Causes of donor death differed between the two cohorts (*p* < 0.001), with a disproportionately higher percentage of deaths in the TPD group attributed to stroke/cerebrovascular accident (21.6%) compared to the Toxoplasma-negative group (14.5%), which may be explained by the known association between toxoplasma and stroke [4]. TPD were more likely to be seen in adult Tx recipients (8.5% versus 5.3%, *p* < 0.0001) and in men (8.7% versus 8.0%, *p* 0.005). Recipient race-ethnicity and causes of CKD were comparable between the two groups. Approximately 9% of each cohort received pre-emptive Tx; HLA mismatches were similar in both groups. Tx from TPD donors occurred in each of the years under study and accounted for approximately 8%–8.5% of all Tx; the largest number of TPD Tx as a fraction of the total Tx, occurred in 2018 (791 of 8,057; 8.9%). Each of the OPTN regions performed TPD Tx with some regional differences (*p* < 0.0001); the majority of TPD Tx occurred in region 3 (n 888) and accounted for 11.3% of all Tx in that region and 20.6% of all TPD Tx nationally. As a fraction of all Tx, region 5 had the lowest number of TPD Tx (5.8%). Delayed graft function was slightly more common in the TPD group (29.9% versus 28.4%, *p* 0.03) but the rates of AR (5.2% in TPD Tx versus 5.0%, *p* 0.51) and 1-year graft failure (5.3% in TPD Tx versus 4.9%, *p* 0.20) were similar between the groups.

**TABLE 2:** Comparing demographic and Tx-related data by Toxoplasma antibody status.

Variables	Toxoplasma IgG status		*p*-value
	Positive (n 4,317)	Negative (n 47,105)	
Recipient age (n, %) Pediatric (<18 years) Adult (18–50 years)	116 (5.3%) 4,201 (8.5%)	2050 (94.7%) 45,055 (91.5%)	<0.0001
Recipient sex (%) Female Male	8.0% 8.7%	92% 91.3%	0.005
Recipient ethnicity (%) Non-Hispanic White Hispanic Black	8.1% 8.1% 8.8%	91.9% 91.9% 91.2%	0.29
Causes of CKD (%) Glomerular Structural	78.0% 11.2%	77.2% 11.3%	0.27
Donor cause of death (%) Anoxia Head trauma Cerebrovascular/stroke	43.3% 31.4% 21.6%	52.4% 29.3% 14.5%	<0.001
Pre-emptive Tx (%)	9.9%	9.8%	0.89
HLA mismatch (%) 0 3 6	4.7% 13.3% 15.1%	4.1% 14% 14.9%	0.31
DGF (%)	29.9%	28.4%	0.03
Treatment for rejection at 1 year (%)	5.2%	5.0%	0.51
1 year graft failure rate	5.3%	4.9%	0.20

CKD, chronic kidney disease; DGF, delayed graft function; Tx, Transplant; HLA, human leukocyte antigen.

Unadjusted graft failure rates per 100 patient-years of follow-up are depicted in Table 3 and were significantly different in the two cohorts with a higher rate in recipients of TPD organs (7.4/100 patient-years in TPD Tx versus 6.5/100 patient-years, *p* 0.008), and a failure rate ratio of 1.14 (95th percentile confidence interval of 1.03, 1.25). No differences were noted in the pediatric-only cohort with a failure rate ratio of 1.03 (95th percentile confidence interval of 0.38, 2.82) in the TPD Tx cohort (*p* 0.95).

**TABLE 3:** Unadjusted Graft failure Rates by donor Toxoplasma status.

	Failure rate per 100 patient-years	95th% confidence intervals	*p*-value
Toxoplasma positive	7.4	6.7, 8.1	0.008
Toxoplasma negative	6.5	6.3, 6.7	
Rate ratio	1.14	1.03, 1.25	

### Graft Failure

On multivariate regression analyses, several independent predictors of graft failure, previously described, were noted (see Table 4). These included recipient sex (higher risk of failure in males), recipient age, with the oldest three recipient age groups in the study having the highest risk of graft loss compared to the youngest group (0–11 years) (adjusted hazard ratio for the 50+ year cohort compared to the 1–11 years group 2.7, 95th percentile confidence interval 1.9, 3.8; *p* < 0.001), recipient ethnicity with white and Hispanic recipients having a lower risk of graft loss compared to Black ethnicity (adjusted hazard ratio for graft loss in the Hispanic cohort 0.80; *p* < 0.001), receipt of pre-Tx dialysis (hazard ratio 1.59, compared to pre-emptive Tx), pre-Tx serum hypoalbuminemia (hazard ratio 0.63 in the cohort with a serum albumin >3.5 g/dL compared to those with serum albumin <2.5 g/dL), increasing donor age (for every 1 year increase in donor age, the hazard ratio for graft loss increased by 1.009; *p* < 0.0001), 5+ HLA mismatches and CIT (for every 1 h increase in CIT, the hazard ratio for graft loss increased by 1.01; *p* < 0.001). Donor Toxoplasma antibody status was not a significant predictor of graft failure (adjusted hazard ratio for TPD Tx 1.04, 95th percentile confidence interval 0.95, 1.15; *p* 0.39). This was also true in the pediatric-only cohort (adjusted hazard ratio for graft failure for TPD Tx 0.66, 95th percentile confidence interval 0.23, 1.91; *p* 0.45).

**TABLE 4:** Multivariate analyses on key predictors of graft loss.

Predictor variable	Adjusted hazard ratio for graft loss	95th% confidence intervals	*p*-value
Recipient sex (male)	1.07	1.01, 1.14	0.02
Recipient age (compared to 1–11 years) 12–18 years 19–32 years 25–50 years 50+ years	1.47 2.38 1.52 2.27	0.96, 2.25 1.55, 3.64 1.06, 2.19 1.86, 3.83	0.16 0.08 <0.001 0.02 <0.001
Recipient race-ethnicity (Black) White Hispanic	0.89 0.81	0.83, 0.96 0.74, 0.88	0.001 <0.0001
Donor age	1.009	1.007, 1.012	<0.0001
Receipt of pre-Tx dialysis	1.59	1.41, 1.79	<0.0001
Serum albumin at Tx (compared to <2.5 g/dL) 2.5–3.4 g/dL 3.5 + g/dL	0.64 0.63	0.50, 0.83 0.49, 0.81	0.0007 0.0003
HLA mismatches (Compared to zero) 5 6	1.25 1.24	1.06, 1.46 1.05, 1.47	0.006 0.01
Donor Toxoplasma status (positive versus negative)	1.04	0.95, 1.15	0.39
CIT	1.01	1.01, 1.01	<0.0001

CIT, cold ischemia time; HLA, human leukocyte antigen; Tx, Transplant.

### Causes of Death/Graft Failure

For patients receiving organs from Toxoplasma-negative donors, we observed 3,435 deaths in 69,739 years of follow-up, for a crude rate of 4.93 deaths per 100 years of follow-up (95% CI: 4.76, 5.09). For recipients of TPD organs, we observed 357 deaths in 6,355 years of follow-up, a crude rate of 5.64 deaths per 100 years of follow-up (95% CI: 5.06, 6.23). The unadjusted rate ratio was 1.14 (1.02, 1.27); *p* = 0.02. However, when we adjusted the estimate, using the same covariates as were used to model graft loss, the adjusted rate ratio was 1.02 (0.91, 1.24); *p* = 0.73. Secondary outcomes were infection as the cause of death, infection as the cause of graft failure and a composite of infection as the cause of either death or graft failure, compared to all other known causes of death. As shown in Table 5, there were no statistically significant differences in any of these secondary outcome measures between the Toxoplasma-negative and TPD cohorts.

**TABLE 5:** Infection as a cause of death or graft loss.

	Infection as a cause of death # (% of all deaths in a row)	Infection as a cause of graft loss # (% of all graft losses in a row)	Infection as a cause of graft loss or death (n, % of all losses + deaths in row)
Toxoplasma positive	153 (58.2%)	15 (8.3%)	167 (40.1%)
Toxoplasma negative	1,420 (55.2%)	124 (7.5%)	1,506 (37.7%)
Toxoplasma positive versus negative row percentage difference (95% Miettinen-Nurminen Confidence Interval)	2.9 (−3.3, 9.1)	−0.8 (−2.7, 5.9)	2.5 (−3.6, 7.4)
*p*-value	0.36	0.70	0.33

## Discussion

Based on our study, the largest to date exploring the outcomes of renal Tx recipients categorized by donor Toxoplasma status, we can reasonably recommend that TPD Tx is safe to perform with close monitoring, and that such organs should not be reflexively discarded, with the caveats discussed below. We would like to note here that we are not aware of any published data on whether Tx centers routinely use or discard TPD kidneys and how, if at all, they decide to triage such organs. This is a gap in our current understanding but based on the experience at our own transplant center and those in our immediate region, we know that there is significant center variation among centers and that some centers have varying degrees of concern about accepting such organs. Our analyses demonstrate that when adjusted for other covariates known to be associated with graft survival, TPD Tx had comparable survival to those from Toxoplasma-negative donors. While this was not statistically significant, the confidence intervals suggest that graft failure in recipients of TPD Tx could be as much as 5% lower to as much as 15% higher, compared to Toxoplasma-negative donor Tx. Since the majority of cases of donor-derived Toxoplasmosis would be expected to occur shortly after Tx [6], it is unlikely that longer follow-up would yield different results. However, whether a larger sample size would change the results is worth exploring through ongoing studies, especially prospective studies of TPD Tx recipients.

From our study it was encouraging to note that Tx from TPD occurred in all OPTN regions and in each year under study with some geographic and temporal variations that are of unclear significance but may represent geographic differences in Toxoplasma seropositivity in the United States (US) based on sociodemographic factors [7] and unique practices and preferences of centers in accepting such donors. However, the number of such Tx is quite small, both in absolute numbers and as a fraction of all Tx, accounting for only 8.4% of all the Tx during our study period. This compares to a prevalence of Toxoplasma of approximately 11% in the general US population [8] and a prevalence of 17.2% in renal Tx donors based on single-center studies [9].

Patients who are seronegative for Toxoplasma and received TPD organs have been noted to have higher seroconversion rates [6] and, while infrequent, also develop life-threatening and fatal infectious complications [6, 10, 11], especially in heart Tx recipients [11]. In the current era, based on the routine use of trimethoprim-sulfamethoxazole (TMP-SMX) prophylaxis in the post-Tx period in all patients to prevent Pneumocystis, a drug that is also effective against Toxoplasma [12], some have suggested not even checking Toxoplasma antibody status in non-heart Tx recipients and in geographic areas with a low Toxoplasma seroprevalence [9] while others have recommended close monitoring and follow-up [6]. Even in the setting of post-Tx Toxoplasmosis infections, outcomes have been favorable with early detection and treatment, even in the highest risk groups [9, 11, 13], further justifying the use of TPD for Tx. Further supporting our recommendations was our observation that infections as a cause of death or graft loss (or a composite of both) were not significantly higher in the TPD cohort, as might be expected if the Toxoplasma positivity were expected to have a detrimental effect on survival.

The limitations of our study pertain to the limited data available in the OPTN database. These include restriction of analyses to recipients of DD renal Tx only (since testing for Toxoplasma is neither required by nor reported to the OPTN), lack of availability of recipient Toxoplasma status to assess donor-recipient mismatch (although based on the aforementioned literature, this may not be as relevant), and the use of antimicrobial prophylaxis in the post-Tx period. We acknowledge that there may be selection bias introduced, since Tx centers may selectively opt for Tx TPD organs in seropositive recipients as they are at lower risk of post-Tx toxoplasmosis. To account for this to the best of our ability, we analyzed outcomes in a pediatric-only subset of Tx recipients. Children are more likely to be Toxoplasma naïve and therefore at the greatest risk of developing post-Tx complications from Toxoplasmosis. We did not find any differences in outcomes in this population, which is reassuring.

In spite of these limitations, and in support of the smaller studies discussed above, our data confirm that Tx from TPD occur in all geographic regions of the US and are associated with comparable graft failure rates. We do strongly advocate for ongoing donor testing for Toxoplasma, testing of Tx recipients for Toxoplasma, universal TMP-SMX prophylaxis if either the donor or recipient is positive, and close monitoring of patients, especially after discontinuation of prophylaxis, as late-onset Toxoplasmosis may occur [10, 11]. Based on our data, we suggest that Tx centers re-evaluate their current policy on the acceptance of TPD organs in light of recent data, and not discard such organs without considering the pros and cons of doing so, for each individual potential Tx recipient. Even if all of our recipients were Toxoplasma seropositive (which is unlikely), we believe that this study adds to the literature and would be of practical value and benefit in that at least in the recipient cohort that is seropositive for Toxoplasma, the use of TPD organs should not be a cause for concern.

## Data Availability

The data analyzed in this study is subject to the following licenses/restrictions: Datasets are only available to member organizations. Requests to access these datasets should be directed to https://optn.transplant.hrsa.gov/data/about-data/optn-database/.

## Footnotes

1
https://unos.org/news/guidance-regarding-donor-toxoplasma-screening-and-organ-acceptance/

## Abbreviations

AR, Acute rejection; HLA, Human Leukocyte Antigen; CIT, Cold ischemia time; CKD, Chronic kidney disease; DD, Deceased Donor; DGF, Delayed graft function; OPTN, Organ Procurement and Transplantation Network; TPD, Toxoplasma-positive donor; Tx, Transplantation.
